# Effect of Oxalic Acid Treatment on Conductive Coatings Formed by Ni@Ag Core–Shell Nanoparticles

**DOI:** 10.3390/ma15010305

**Published:** 2022-01-01

**Authors:** Anna Pajor-Świerzy, Radosław Pawłowski, Piotr Sobik, Alexander Kamyshny, Krzysztof Szczepanowicz

**Affiliations:** 1 Jerzy Haber Institute of Catalysis and Surface Chemistry Polish Academy of Sciences, Niezapominajek 8, 30-239 Krakow, Poland; krzysztof.szczepanowicz@ikifp.edu.pl; 2 Abraxas Jeremiasz Olgierd, Piaskowa 27, 44-300 Wodzisław Śląski, Poland; radek.pawlowski@helioenergia.com (R.P.); piotr.sobik@helioenergia.com (P.S.); 3 Casali Center for Applied Chemistry, Institute of Chemistry, Edmond J. Safra Campus, The Hebrew University of Jerusalem, Jerusalem 91904, Israel; alexander.kamyshny@mail.huji.ac.il

**Keywords:** nickel–silver core–shell nanoparticles, conductive coatings, sintering, conductivity, oxalic acid

## Abstract

Low-cost metallic nanoink based on nickel–silver core–shell nanoparticles (Ni@Ag NPs) was used for the formation of conductive metallic coatings with low sintering temperature, which can be successfully applied for replacement of currently used silver-based nanoinks in printed electronics. The effect of oxalic acid (OA) on the sintering temperature and conductivity of coatings formed by Ni@Ag NPs was evaluated. It was found that the addition of OA to the ink formulation and post-printing treatment of deposited films with this acid provided a noticeable decrease in the sintering temperature required for obtaining conductive patterns that is especially important for utilizing the polymeric substrates. The obtained resistivity of metallic coatings after sintering at temperature as low as 100 °C was found to be 30 µΩ·cm, only ~4 times higher compared to the resistivity of bulk Ni that is promising for future application of such materials for fabrication of low-cost flexible printed patterns.

## 1. Introduction

Printed electronics is a growing field in applied materials science and technology, among which flexible printed electronics, referring to printed devices that are stable under bending, twisting, and folding, is a major trend [1]. To fabricate flexible printed electronic devices, the optimal selection of materials for the formulation of conductive inks and flexible substrates, and the methods selected for printing and post-printing treatment are of crucial importance. Flexible printed electronics require a selection of flexible substrates, and the most widely used include various polymers such as polyethylene terephthalate (PET), polyimide (PI), and polyethylene naphthalate (PEN), as well as papers and textile fabrics [2,3,4,5]. The main challenge in the use of flexible substrates for printed electronics is their low thermal stability, while obtaining high electrical conductivity in printed circuits usually requires sintering at elevated temperatures [2,3,6,7,8,9,10]. This process removes the insulating organic components of the ink and allows the welding of metallic NPs, in order to form a continuous electrical network [2,3]. For the most popular flexible substrates, the temperature of sintering (or any post-printing processing) should be no higher than 150 °C [1]. Therefore, the development of alternative sintering methods focusing mainly on avoiding the destruction of the flexible substrate and enabling fast and efficient fabrication of conductive coatings has attracted great interest in recent years [11,12]. As promising approaches, chemical- [13,14] and radiation-induced [15,16] sintering methods have been considered. 

In recent years, carboxylic acids have attracted much attention regarding their application in the process of synthesizing metallic NPs [17,18], as well as in the fabrication of conductive structures [17,19,20,21], due to their low decomposition temperature, and antioxidation and reductive capabilities. The effect of organic acids on the properties of copper NPs and the conductivity of copper coatings have been reported by Deng et al. [17]. It was found that using acetic, glycolic, and lactic acid as capping agents led to a decrease in the oxidation degree of the NPs because of the presence of densely packed organic material on the copper surface of copper NPs. The obtained metallic films were conductive after sintering at temperatures as low as 150 °C. A minimum resistivity value of 21 µΩ·cm was obtained for metallic coatings based on Cu NPs synthesized in the presence of lactic acid as a capping agent. Kim et al. [19] obtained Cu NP interconnectors with low resistivity (~72 µΩ·cm) after sintering under a formic acid atmosphere at 150 °C. This low sintering temperature was primarily due to the decomposition of the capping molecules covering the surface of the NPs and the reduction of the copper oxide by formic acid. Similarly, the reduction effect of various types of carboxylic acids (formic, acetic, propionic, butyric, oxalic, and citric acid) has been demonstrated on inkjet-printed Cu coatings [20]. In our previous research, we studied the effect on the conductivity of deposited metallic coatings of adding selected carboxylic acids to the Cu-Ag ink, and revealed increased conductivity for all tested acids, with the highest effect being found for oleic acid [21]. Among carboxylic acids, dicarboxylic OA shows reducing [20,22], complexing [23,24,25], and antioxidative [26,27] properties for various types of metals. Therefore, in the present work, we focused on the effect of OA on the conductive properties of metallic coatings based on Ni@Ag NPs. As was reported recently [6,7,8,9,28], Ni@Ag NPs can be successfully used as conductive components of inks for printed electronics and as a cheaper alternative for silver. The effect of the addition of OA to the Ni@Ag ink as well as post-printing treatment on the conductivity of printed coatings was investigated. We found that Ni@Ag coatings after post-printing treatment with an optimal concentration of OA are promising for the fabrication of flexible printed electronics. 

## 2. Materials and Methods

### 2.1. Materials 

Nickel sulfate hexahydrate (NiSO_4_·6H_2_O), sodium borohydride (NaBH_4_), sodium carboxymethyl cellulose (NaCMC) with MW 90000, silver nitrate (AgNO_3_), citric acid (CA), oxalic acid, and aminomethyl propanol (AMP) were purchased from Sigma-Aldrich (Poznań, Poland). Surfynol PSA 336 (acetylenic-based formulated surfactant, wetting agent) was a product of Evonik (Essen, Germany).

### 2.2. Fabrication of Ni@Ag NPs Based Ink

Ni@Ag NPs were synthesized by using a two-stage process, according to the method presented in our previous reports [9,28]. An aqueous dispersion of Ni NPs was obtained by reduction of Ni ions with NaBH_4_ in the presence of two complexing agents (CA and AMP) and NaCMC as a stabilizing agent. First, 30 mL of 0.5% CMC, 12 mL of 0.2 M NiSO_4_, and 12 mL of 0.15 M CA were mixed, and an 80% solution of AMP was added to adjust pH value to 9. Then, by using a peristaltic pump (700 rpm), 30 mL of NaBH_4_ aqueous solution with a concentration of 0.05 M was injected into the reaction mixture followed by stirring at 850 rpm for 60 min. The silver shell was formed on the surface of synthesized Ni NPs by transmetalation (displacement) reaction. This reaction was performed by addition of 45 mL of the aqueous solution of AgNO_3_ (0.04 M) as a silver shell precursor to the dispersion of Ni NPs and stirring for 60 min at room temperature. The obtained Ni@Ag NPs were washed with distilled water to remove an excess of stabilizing agent and other additives and concentrated to 25 wt% by centrifugation/redispersion (two times).

The ink formulation was performed as described in [9]. More specifically, Surfynol PSA 336 (0.025 wt%) was added as a wetting agent to the concentrated (25 wt%) dispersion of Ni@Ag NPs followed by ultrasonication (30 min at 20 kHz) to obtain homogeneous ink. To study the effect of OA on the ink formulation as well as on a final conductivity of coatings, various concentrations of OA (in the range of 0–2%) were added to the ink formulation, and the obtained dispersion was homogenized again (ultrasonic bath, 30 min at 20 kHz).

### 2.3. Metallic Coating Fabrication

The ink was deposited on glass slides (3.5 cm × 2.5 cm) by bar coating with the use of K-Hand Coater (Kontech, Łódź, Poland) [29]. After coating, the ink layers were dried on a hot plate at 40 °C for 15 min. To study the effect of post-printing treatment with OA, the deposited coatings were dipped for one minute into the aqueous solutions of OA at various concentrations (0–2%), and then treated films were dried on a hot plate at 40 °C for 15 min and sintered by heating at a temperature in the range of 80–200 °C for 15–75 min. 

### 2.4. Characterization 

The size of Ni@Ag NPs was measured by dynamic light scattering (DLS), and the zeta potential with the microelectrophoretic method using Zetasizer Nano Series (Malvern Instruments). Each value was measured in three runs with at least 20 measurements at 25 °C. The ink coatings were obtained by using a hand coater (Kontech, Łódź, Poland). Their topography and morphology were visualized using an optical microscope (Hirox, HR-2500) and by scanning electron microscopy (SEM, LEO Gemini 1530, Zeiss, Jena, Germany). The chemical composition of sintered coatings based on Ni@Ag NPs before and after treatment with OA was evaluated by X-ray photoelectron spectroscopy (XPS) with an ESCA/XPS equipped with a semispherical analyzer EA15 (Prevac) using Al-Kα (1486.6 eV) radiation with a power of 180 W. The thickness of deposited films was measured by the EDXRF method (FISCHERSCOPE X-RAY XDL 230, Worcestershire, UK). The obtained coatings were sintered by heating on a hot plate in an atmospheric environment. The sheet resistances of metallic films were determined using a four-point probe method (Milliohm Meter, Extech Instruments, Nashua, NH, USA). In this technique, four equally spaced, co-linear Calvin probes were manually contacted with the coated films, resulting in electrical contact, and the values of the sheet resistance were automatically measured [30]. The resistivities of metallic coatings were calculated by multiplying the measured the sheet resistances by the thicknesses of the films [31].

## 3. Results and Discussion

**Figure 1:**
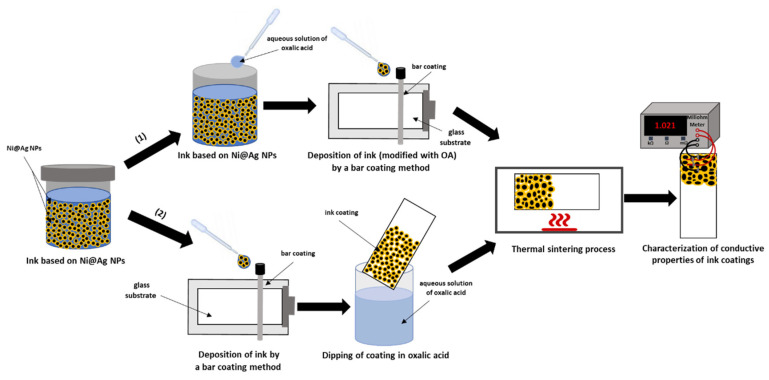
Scheme of the preparation of the conductive coatings based on Ni@Ag NPs: (1) with addition of oxalic acid to ink formulation; (2) after post-printing treatment with OA.

In general, two steps are required to obtain conductive patterns when using metallic nanoinks: their deposition using a proper coating method, and post-coating sintering, which results in the formation of a continuous metallic film of tightly packed or welded NPs that do not contain insulating organic additives. In the present research, two approaches were used to study the effect of OA on the conductivity of films formed by Ni@Ag ink: (1) addition of OA to the ink, followed by its deposition on the substrate and sintering; and (2) post-deposition treatment with OA before sintering (Figure 1).

### 3.1. Fabrication of Ni@Ag NP-Based Ink

**Figure 2:**
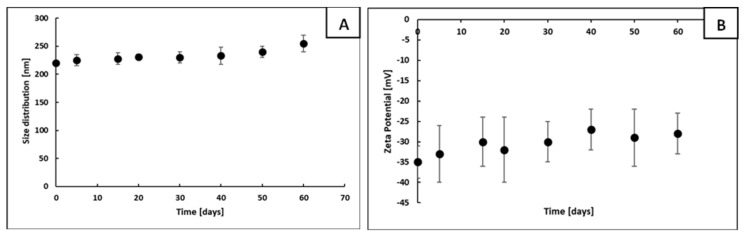
Dependence of size (**A**) and zeta potential (**B**) of Ni@Ag NPs on the storage time measured by DLS and by microelectrophoretic method, respectively.

The Ni@Ag NP-based ink was prepared according to the method developed in our lab and reported recently [9,28]. The synthesized Ni@Ag NPs had an average size of 220 nm and a zeta potential of −35 mV (Figure S1). These characteristics of Ni@Ag NPs remained practically unchanged for at least 60 days, which indicates their stability in aqueous dispersion (Figure 2). A detailed characterization of such NPs can be found in our recent papers [9,28]. To optimize the properties of ink coatings, a wetting agent, Surfynol PSA 336, was added, and the obtained formulation, which contained 25 wt% of NPs, was used as metallic ink.

### 3.2. Effect of OA Addition on the Properties of Ni-Ag-Based Ink 

**Figure 3:**
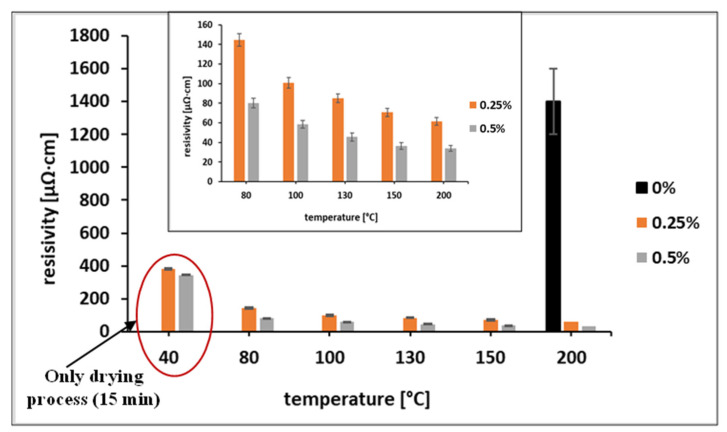
Resistivity of coatings formed by Ni@Ag NPs inks containing 0.25 and 0.5 wt% OA as a function of sintering temperature in the range of 40–200 °C (heating duration 30 min). Black bar represents resistivity of ink coatings without OA.

According to previous findings, the addition of selected carboxylic acids to Cu and Cu@Ag NP-based inks results in effective protection of the NPs from oxidation, as well as noticeable improvement in the electrical characteristics of the metallic coatings [17,19,20,21]. After the addition of OA to the ink at concentrations of 1 wt% or higher, visually significant agglomerations of Ni@Ag NPs were observed. We assume that OA causes the detachment of the anchoring groups of the stabilizing polymer from the surface of the NPs by adsorbing the OA molecules or by decreasing the pH (from about 8 to 5.5) and through desorption of protonated CMC molecules from the surface of Ni@Ag NPs. This effect was demonstrated for the CMC layer adsorbed on the surface of Ag NPs at pH lower than 4.4 [32]. A similar “destabilizing agent” effect (NaCl), causing desorption of polyacrylic acid from Ag NPs along with their agglomeration, has been described previously [13]. As OA does not cause aggregation and agglomeration at concentrations lower than 1 wt%, further experiments were performed with inks containing 0.25 and 0.5 wt% OA. These inks were deposited on glass slides by bar coating and sintered at a temperature in the range of 80–200 °C. The dependence of the coating resistivity on the sintering temperature (heating duration 30 min) is presented in Figure 3. As seen, the addition of OA to the ink formulation significantly affected the conductivity of the obtained metallic coatings. The measurable resistivity values for the ink coatings that did not contain OA were obtained only after sintering at 200 °C, while coatings containing OA were found to be conductive even after sintering at temperatures as low as 80 °C. In addition, decreases in resistivity from 145 to 61 µΩ·cm (~8.7 times higher compared to the resistivity of bulk Ni) and from 80 to 34 µΩ·cm (~4.9 times higher compared to the resistivity of bulk Ni) was observed for ink coatings containing 0.25% and 0.5% of OA, respectively, when increasing the sintering temperature from 80 to 200 °C.

### 3.3. Post-Printing Treatment with OA

**Figure 4:**
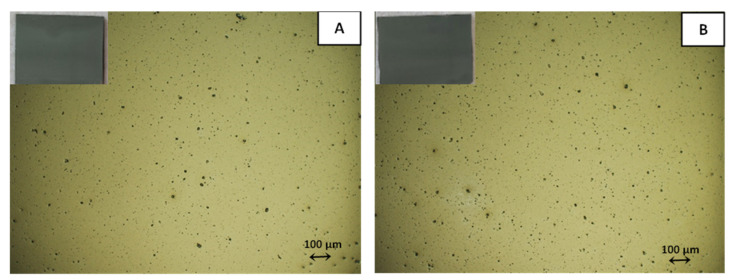
Examples of optical microscopy images of ink coatings after drying (15 min, 40 °C) before (**A**) and after (**B**) treatment with 1% OA.

Another approach for studying the effect of OA on the conductivity of Ni@Ag coatings was their post-printing treatment with oxalic acid. Ink containing Ni@Ag NPs was deposited on glass slides using the same method (bar coating), and then the deposited coatings were dried on a hot plate for 15 min at 40 °C in ambient atmosphere and dipped (for 1 min) in aqueous solutions of OA with concentrations in the same range, 0–2% wt%, and dried again under the same conditions. The morphology of the coatings was evaluated by an optical microscope to ensure good quality, which is an important characteristic for obtaining high conductivity. As seen from Figure 4, there were no cracks and noticeable defects on the surface of the coatings before or after treatment with 1 wt% solution of OA (only small holes were noticed). 

It was also found that even after drying the deposited coatings treated with 1 wt% OA, the resistivity was measured to be 220 µΩ·cm, which confirms the effect of OA on the electrical characteristics of obtained coatings.

**Figure 5:**
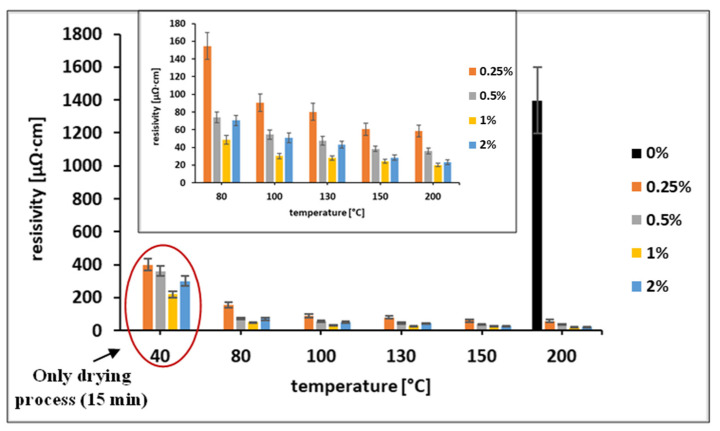
Dependence of the resistivity of the coatings based on Ni@Ag NPs on the concentration of OA (0–2%) after drying (40 °C, 15 min) and sintering (80–200 °C, heating duration 30 min). The inset graph presents the data in the resistivity range of 0–180 µΩ·cm.

Moreover, the films formed by Ni@Ag NPs were sintered at various temperatures ranging from 80 to 200 °C. The thicknesses of all of the sintered films were similar to those obtained after drying, about 2 µm, as seen in Figure S2. The dependence of the resistivity of the metallic films on the concentration of OA after drying (40 °C, 15 min) and sintering (80–200 °C, heating duration 30 min) is presented in Figure 5.

**Figure 6:**
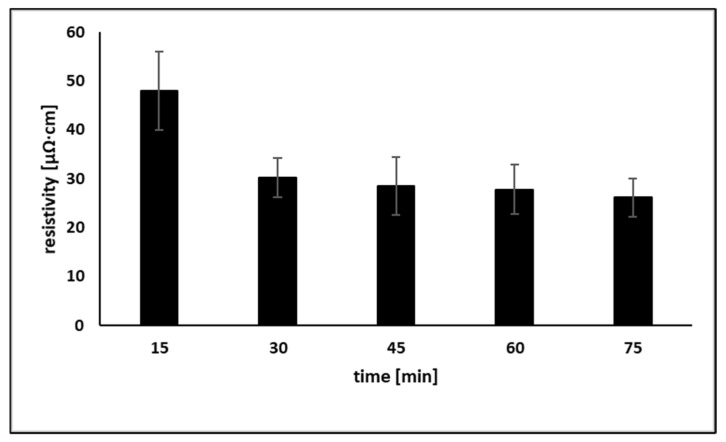
Dependence of the resistivity of Ni@Ag NP coatings treated with 1% OA on sintering duration (15–75 min) at sintering temperature 100 °C.

It was found that the sheet resistance of the coatings that were not treated with OA was too high for detection after sintering at temperatures below 150 °C. After sintering at 200 °C, the resistivity was still very high (black bar in Figure 5). However, the resistivities of samples treated with OA and sintered at 80 °C were significantly lower at all OA concentrations (0.25–2 wt%) as compared with non-treated samples sintered at 200 °C. Based on the obtained data, post-printing treatment with 1 wt% OA was selected as the optimal treatment. This treatment enables a decrease of the sintering temperature to 100 °C, which is acceptable for the sintering of metallic coatings on flexible substrates like polymer films, paper, or textiles. In addition to temperature, the sintering duration is also an important parameter when fabricating conductive coatings. As was found (Figure 6), the optimal duration of sintering at 100 °C is in the range of 30–75 min. 

The resistivity of coatings sintered for 30 min was 30 µΩ·cm, only about 4 times higher than the resistivity of bulk Ni. Furthermore, increasing the sintering duration has practically no effect on resistivity. Therefore, 30 min duration and 100 °C were selected as optimal conditions for obtaining conductive Ni@Ag coatings on a glass substrate treated with 1 wt% OA. To the best of our knowledge, this is the first time such low resistivity at a sintering temperature as low as 100 °C for coatings based on Ni and Ni@Ag NPs has been obtained. In our previous report [9], we showed that films composed of Ni@Ag NPs doped with 1% of Ag NPs had a slightly lower resistivity (35% of bulk nickel), but at a higher sintering temperature, 150 °C, which is a limiting temperature for polymeric films as flexible substrates. Moreover, using the lower sintering temperature is more profitable from an economic point of view.

### 3.4. Mechanism of OA Action

**Figure 7:**
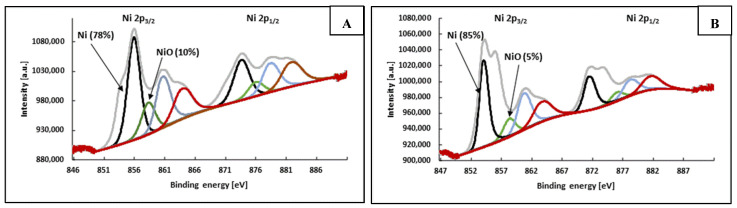
XPS of Ni@Ag NP coatings (after sintering at 100 °C for 30 min) without OA treatment (**A**) and after treatment with 1 wt% OA (**B**) (metallic Ni—black curves, NiO—green curves, Ni^2+^—red curves, satellites—blue curves.

Although the improved conductivity of the coatings formed by Ni@Ag NP-based ink is clearly demonstrated, the mechanism of OA action seems to be neither unequivocal nor trivial, and several possibilities should be considered. OA is the simplest dicarboxylic acid, and can be used as a reducer, complexing agent, or antioxidant [20,23,24,25,26,27]. OA is also a well-known rust remover capable of dissolving nickel oxide, which can be formed on the surface of NPs. As was assumed by Figueroa et al. [33], the mechanism of OA action involves the transfer of nickel oxide to two different soluble surface complexes with this acid and their dissolving to nickel ions, which could be transformed into metallic nickel during the thermal sintering process, resulting in the improved conductivity of the obtained coatings. The formation of three types of OA complexes with nickel ions—[NiHL]^+^, [NiL], [Ni(OH)L]^−^, and [NiL_2_]^2−^—was also reported by Penuela et al. [25]. Another explanation for the mechanism of action of OA is related to its redox properties (E^0^ = −0.49), since OA is capable of reducing nickel oxide. It was observed that among various carboxylic acids (formic, acetic, propionic, butyric, oxalic, and citric), OA was the most effective as a reducing agent in the process of sintering the film formed by Cu NPs, and OA treatment resulted in obtaining films with low resistivity [20]. Kanzaki et al. [26] prepared conductive coatings based on Cu NPs ink containing 1% OA, with a resistivity of 5.5 × 10^−5^ Ω·cm at thermal sintering (150 °C). The mechanism of sintering was explained as the ability of OA to protect Cu NPs from oxidation. The same effect can take place in the case of Ni@Ag NPs. Figure 7 presents the XPS analysis of Ni@Ag NP coatings without OA treatment and treated by OA.

**Figure 8:**
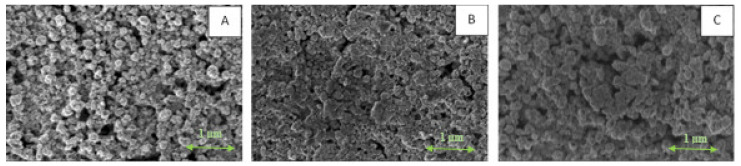
SEM images of Ni@Ag coatings after drying (**A**); after drying, dipping in OA (1 wt%) and drying (40 °C) (**B**); after drying, dipping in OA (1%) followed by drying and sintering (100 °C, 30 min) (**C**).

As seen in Figure 7, after sintering of the coating not treated with OA (Figure 7A), the atomic percentage of NiO is two times higher in comparison to the one obtained under the same sintering conditions after treatment with 1 wt% OA (Figure 7B). This result may caused by both the improved protection of Ni@Ag NPs against oxidation and the reduction/dissolution of NiO by OA. Moreover, as previously indicated, OA can also act as a “destabilizing agent”, which was clearly observed after the addition of OA to the dispersion of Ni@Ag NPs. As the organic stabilizer would be removed from the surface of NPs, the formation of their aggregates may cause the transformation of the crystalline structure, leading to sintering taking place that is similar to that occurring at high temperature [13,34]. SEM analysis confirmed the effect of OA on the morphology of metallic coatings based on Ni@Ag NPs. As seen in Figure 8, the NPs in the coating after treatment with 1% wt% OA and drying at 40 °C for 15 min (Figure 8B) and then sintered at 100 °C (Figure 8C) were much more tightly connected or even welded as compared with a coating not treated with OA (Figure 8A), which exhibited a noticeable decrease in the resistivity of the coating. Therefore, we conclude that some kind of chemical sintering [1,2,13], as a result of OA action, also takes place, resulting in metallic coatings with enhanced conductivity being obtained at low-temperature sintering.

## 4. Conclusions

The treatment of metallic coatings based on Ni@Ag NPs with OA results in lowering the sintering temperature to 100 °C, with the formation of metallic substances with good conductivity. A possible mechanism of the OA action is the chemical sintering of Ni@Ag NPs by destroying an insulator layer of stabilizer combined with dissolution/reduction of the formed nickel oxides or complex formation. Post-printing treatment with OA would allow the application of Ni@Ag NP-based coatings for low-sintering temperature processes, which are crucial for the fabrication of flexible printed electronic devices. The next step in our research will be adapting this technology to thermosensitive flexible substrates.

## Data Availability

Not applicable.

## Supplementary Materials

The following are available online at https://www.mdpi.com/article/10.3390/ma15010305/s1, Figure S1: Size distribution (**A**) and zeta potential (**B**) of Ni@Ag NPs as an average of three subsequent runs. Figure S2: The thickness of the metallic coating treated with 1 wt% OA after sintering as measured by the EDXRF method.
