# Supplementary material for: Effect of Oxalic Acid Treatment on Conductive Coatings Formed by Ni@Ag Core–Shell Nanoparticles
Source: Materials (Basel). 2022 Jan 1;15(1):305. doi: 10.3390/ma15010305 (PMC8746183; doi:10.3390/ma15010305)
Supplement: Supplementary file 1 [file materials-15-00305-s001.zip › materials-1520170-supplementary.pdf]

Supplementary material

Effect of Oxalic Acid Treatment on Conductive Coatings Formed by Ni@Ag Core–Shell Nanoparticles

Anna Pajor-Świerzy [1,*], Radosław Pawłowski [2], Piotr Sobik [2], Alexander Kamyshny [3] and Krzysztof Szczepanowicz [1]

[1] Jerzy Haber Institute of Catalysis and Surface Chemistry Polish Academy of Sciences, Niezapominajek 8, 30-239 Krakow, Poland; krzysztof.szczepanowicz@ikifp.edu.pl
[2] Abraxas Jeremiasz Olgierd, Piaskowa 27, 44300 Wodzisław Śląski, Poland; radek.pawlowski@helioenergia.com (R.P.); piotr.sobik@helioenergia.com (P.S.)
[3] Casali Center for Applied Chemistry, Institute of Chemistry, Edmond J. Safra Campus, The Hebrew University of Jerusalem, Jerusalem 91904, Israel; alexander.kamyshny@mail.huji.ac.il
* Correspondence: anna.pajor-swierzy@ikifp.edu.pl

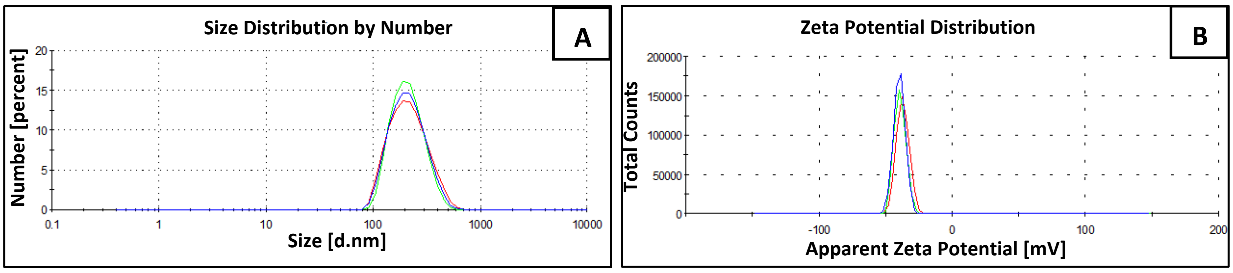

Figure S1. Size distribution (**A**) and zeta potential (**B**) of Ni-Ag NPs as an average of three subsequent runs.

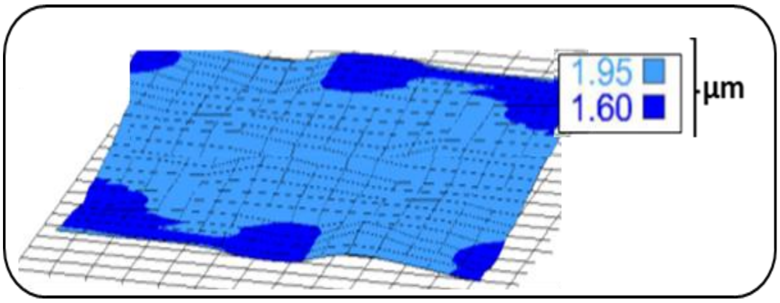

Figure S2. The thickness of the metallic coating treated with 1 wt% OA after sintering as measured by the EDXRF method.
